# Online deployment of an O‐PLS model for dielectric spectroscopy‐based inline monitoring of viable cell concentrations in Chinese hamster ovary cell perfusion cultivations

**DOI:** 10.1002/elsc.202200053

**Published:** 2023-05-17

**Authors:** Johannes Lemke, Robert Söldner, Jonas Austerjost

**Affiliations:** ^1^ Corporate Research Sartorius Stedim Biotech GmbH Göttingen Germany

**Keywords:** dielectric spectroscopy, multivariate data analytics, process analytical technology

## Abstract

Viable cell concentration (VCC) is an essential parameter that is required to support the efficient cultivation of mammalian cells. Although commonly determined using at‐line or off‐line analytics, in‐line capacitance measurements represent a suitable alternative method for the determination of VCC. In addition, these latter efforts are complimentary with the Food and Drug Administration's initiative for process analytical technologies (PATs). However, current applications for online determination of the VCC often rely on single frequency measurements and corresponding linear regression models. It has been reported that this may be insufficient for application at all stages of a mammalian cell culture processes due to changes in multiple cell parameters over time. Alternatively, dielectric spectroscopy, measuring capacitance at multiple frequencies, in combination with multivariate mathematical models, has proven to be more robust. However, this has only been applied for retrospective data analysis. Here, we present the implementation of an O‐PLS model for the online processing of multifrequency capacitance signals and the on‐the‐fly integration of the models’ VCC results into a supervisory control and data acquisition (SCADA) system commonly used for cultivation observation and control. This system was evaluated using a Chinese hamster ovary (CHO) cell perfusion process.

**Abbreviations**
CHO: Chinese hamster ovary
MVDA: multivariate data analysis
O‐PLS: Orthogonal‐Partial Least Squares
PATs: process analytical technologies
RMSEcv: root‐mean‐square error of cross‐validation
RMSEP: root‐mean‐square error of prediction
SCADA: supervisory control and data acquisition
VCC: viable cell concentration

1

The Food and Drug Administration's process analytical technology (PAT) initiative was intended to drive the implementation of efficient monitoring and control strategies of critical process parameters for an improved pharmaceutical manufacturing process and product quality [1]. Here, tools for inline or online monitoring of critical process parameters play a crucial role as they quickly deliver data regarding parameters that are critical to process control.

Among these critical parameters, VCC is determined at‐line or off‐line using imaging of trypan blue exclusion by viable cells [2]. Dielectric spectroscopy systems, commonly known as in‐line capacitance probes, monitor suspension cell concentrations by measuring electrical features of the cells at different radio frequencies [3]. Historically, these measurements and analyses were performed using single‐frequency measurements and linear regression; or the application of the Cole–Cole equation, a dedicated function expressing the permittivity/frequency relationship. Latter studies considered the efficacy of multifrequency measurements and associated multivariate models. Superiority of the latter was demonstrated on historical batch and fed‐batch datasets [4]. Where multivariate models have proven to be robust over the entire process time, linear regression models, as well as Cole–Cole models hold several disadvantages. For example, the need for periodic recalibration during the process and noted challenges with reliability over specific process phases [5, 6]. Nevertheless, single‐frequency measurements and associated linear models are still commonly applied for online process monitoring and control, as the online execution of multivariate models within common process architectures remains a challenge.

**PRACTICAL APPLICATION**

The monitoring of viable cell concentrations (VCCs) is an essential metric for mammalian cell cultivation processes. This parameter is of particular relevance for perfusion cultivation as it is used to adjust the cell bleed rate. The determination of VCC is frequently performed using off‐line assays requiring manual sampling and material consumption; or in‐line capacitance measurement using dielectric spectroscopy. However, common online dielectric spectroscopy approaches rely solely on single‐frequency measurements paired with corresponding linear mathematical models. This approach does not account for the morphological and physiological changes of cells during cultivation. These changes may confound the analysis if unaddressed. In this study, we present the initialization of an online Orthogonal‐Partial Least Squares (O‐PLS) model for processing capacitance scanning data that accounts for these changes. This approach delivers more accurate VCCs than common single‐frequency‐based models. This system represents a robust solution for online dielectric spectroscopy approaches that previously relied solely upon single‐frequency measurements and linear models in perfusion process monitoring and control.

In this study, we present the execution of an online O‐PLS model that is capable of processing the multifrequency data of an in‐line capacitance probe for the robust online determination of VCC in a Chinese hamster ovary (CHO) perfusion process. A flow‐chart of the procedure is depicted in Figure 1. The nascent O‐PLS model was integrated into SCADA software and used to monitor the VCC over all phases of a 26‐day perfusion process. This may represent the initial report of the application of capacitance scanning and a related multivariate model used for the online monitoring of a CHO perfusion cultivation.

**FIGURE 1:**
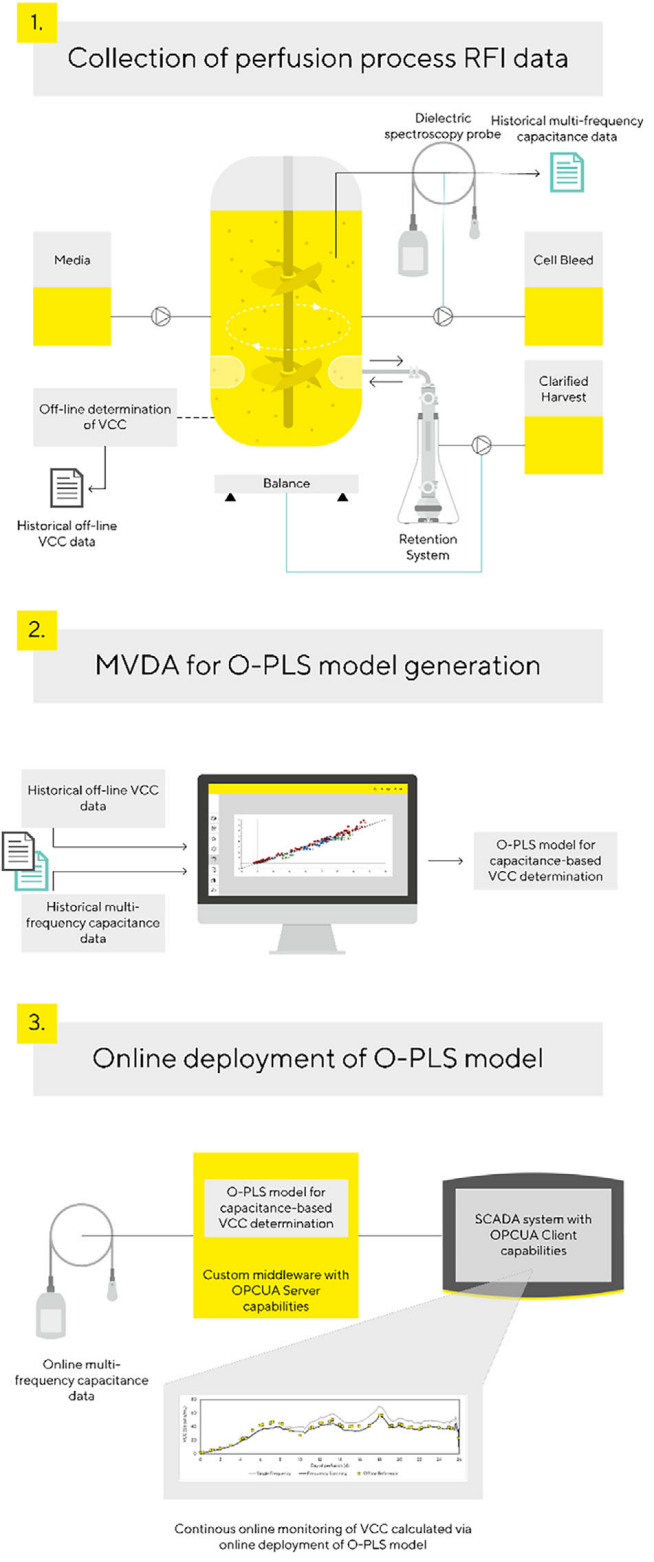
Flowchart depicting the approach for online deployment of an O‐PLS model and the integration into a SCADA system. First, historical multifrequency capacitance data as well as historical off‐line VCC measurement data were collected in several perfusion runs. Second, multivariate data analysis was applied to generate a model that can predict the VCC based on frequency scanning data. Third, a custom IT infrastructure was set up for an online execution of the model and SCADA integration. O‐PLS, Orthogonal‐Partial Least Squares; SCADA, supervisory control and data acquisition; VCC, viable cell concentration.

Perfusion cell cultivation was performed as described by Müller et al. [7]. In brief, an industrial relevant CHO cell line (DG44, Sartorius Stedim Biotech GmbH, Göttingen, Germany) expressing a monoclonal antibody (IgG1) was cultivated using chemically defined medium (XtraCHO SAM and proprietary perfusion medium, Sartorius Stedim Biotech GmbH, Göttingen, Germany). After a 3‐day batch phase in a 2 L Univessel Glass^®^ bioreactors (Sartorius Stedim Biotech GmbH, Göttingen, Germany), perfusion was initiated using an ATF2 device (Repligen, Waltham, MA). Perfusion rate was constantly adapted to maintain a minimum cell‐specific perfusion rate of 50 pL∙cell^−1^∙day^−1^. Automatic cell bleed based on single‐frequency capacitance measurement was started after achieving target cell concentrations utilizing a PID controller in BioPAT^®^ MFCS supervisory control and data acquisition (SCADA) software 4.9 (Sartorius Stedim Biotech GmbH, Göttingen, Germany).

Off‐line samples were taken manually from all cultivations. Cell growth (VCC, viability, and average diameter) were measured with a CedexHiRes Analyzer (Roche Diagnostics GmbH, Mannheim, Germany). The pH, DO, glucose, and lactate levels were measured using a blood gas analyzer (ABL800 Basic, Radiometer GmbH, Krefeld, Germany). The process was stopped after 26 days.

On‐line viable cell concentration was measured using a dielectric spectroscopy probe (BioPAT^®^ Viamass, Sartorius Stedim Biotech GmbH, Göttingen, Germany). The single‐frequency capacitance signal at 580 kHz was correlated to the VCC based on historical data specific for both cell line and medium and applied for process control [8]. Simultaneously, dielectric spectroscopy data were obtained at 25 discrete frequencies between 50 and 20,000 kHz using FUTURA SCADA software (Aber Instruments Ltd, Aberystwyth, UK) and used for online VCC prediction based on an online executed O‐PLS model. Previous studies by Metze et al. showed the successful application of O‐PLS modeling for VCC in fed‐batch processes, which supported our decision of using O‐PLS modeling for perfusion processes [2].

Dielectric spectroscopy data from three different cultivations were collected and used to build an O‐PLS model for VCC prediction. All three perfusion cultivations were conducted using the same cell line and media platform as well as similar process conditions; however, some deviations were included in each run to ensure a suitable data set for robust model building. By implementing a cell bleed with the use of different target VCCs for the cultivations, possible correlations between VCC and batch maturity were reduced. For model building, dielectric spectroscopy data were averaged over 5 min using a rolling mean and merged with the off‐line reference values. Combined data sets were exported to SIMCA 17 (Sartorius Stedim Data Analytics AB, Umeå, Sweden) for further evaluation and model building. The multifrequency capacitance data were mean‐centered and selected as model predictors while VCC reference data were scaled to unit variance and marked as a model response. For the generated O‐PLS model, one predictive and two orthogonal components were used.

To enable the online application of the O‐PLS model, an OPC UA wrapping component (Unified Automation GmbH, Kalchreuth, Germany) was used to make the native OPC DA server component of the FUTURA SCADA software available to OPC UA clients. The custom middleware component for model deployment was developed using Node‐RED 1.3.4 (OpenJS Foundation, San Francisco, CA) [9]. The established custom middleware component consists of an Open Platform Communication Unified Architecture (OPC UA) client component that periodically receives the dielectric spectroscopy data provided by the FUTURA SCADA software. This client component forwards all received data arrays to a function component that incorporates the model equation, which was previously exported using the model export function of SIMCA 17 (Sartorius Stedim Data Analytics AB, Umeå, Sweden). Model results are then exposed via an OPC UA server component. This OPC UA server component and the corresponding online model data were accessed by BioPAT^®^ MFCS version 4.9 as a client and used for process observation purposes.

To quantify the performance of the linear and multivariate capacitance models, the root‐mean‐square error of prediction (RMSEP) was calculated using Equation (1) where ${\hat{y}}_{i}$ describes the predicted VCC value based on the applied model, $y_{i}\;$describes the off‐line reference value and *n* the number of VCC prediction and reference value pairs.

(1) $$RMSE\;=\;\sqrt{\frac{\sum_{i\;=\;1}^{n}{\left({\hat{y}}_{i}-y_{i}\right)}^{2}}{n}}$$

In addition, the prediction capabilities on the data sets used for building the multivariate model were estimated by calculation of the root‐mean‐square error of cross‐validation (RMSEcv). For this cross‐validation, some data pairs were left out of model development and then predicted by the model, which is repeated until all parts have been kept out once.

Figure 2 shows the observed versus predicted VCC plots for both single‐frequency (A) as well as multifrequency capacitance data (B). Three perfusion cell cultivations were performed to generate sufficient data for model building at a broad range of VCCs, up to 80E6 cells/mL. For lower cell concentrations, up to approximately 30E6 cells/mL, both models were able to predict the VCC in the perfusion bioreactor with a high degree of accuracy, demonstrated by the strong linear correlation shown in Figure 2 and B. At higher VCCs, the single‐frequency model demonstrates increased deviations between observed and predicted VCCs, resulting in a RMSEP of 10.8E6 cells/mL for the complete culture duration. In contrast, the O‐PLS model based on multifrequency capacitance data demonstrates much higher accuracy in VCC prediction for the whole range of cell concentrations with a RMSEcv of 3.7E6 cells/mL. The multifrequency model was generated using data from perfusion batches 1–3, whereas the single‐frequency model was generated using historical data from other perfusion batches. Therefore, a comparison of these models is not ideal. In order to enhance the evaluation of their predictive performance, employing an external dataset that is distinct from the one used for model training is recommended. This was done by running both models online in a perfusion run. However, the observed versus predicted plot of the linear model in Figure 2A shows that it predicts values that are either too high or too low. This suggests that even recalibration of the model would not significantly improve its predictive performance.

**FIGURE 2:**
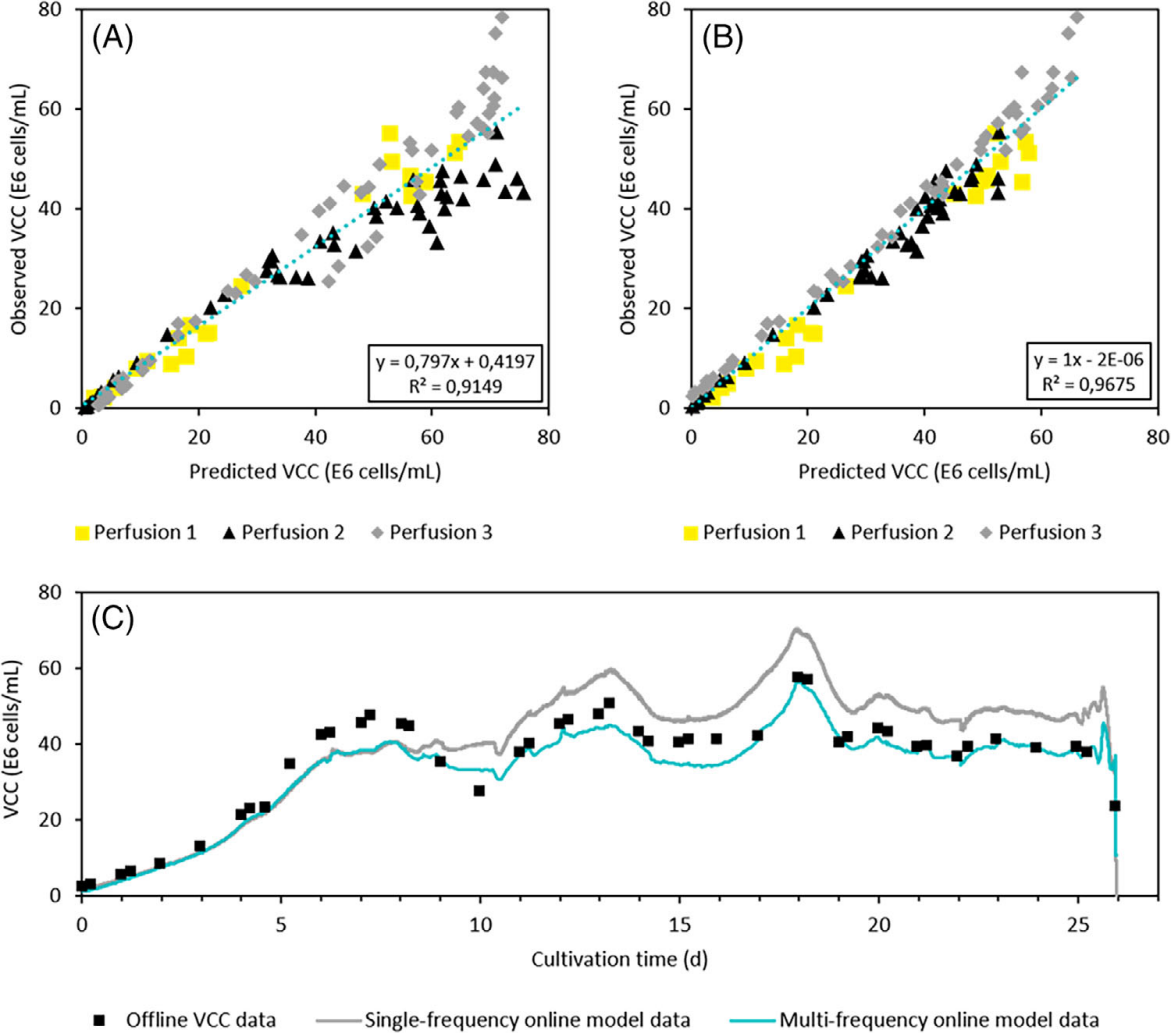
Observed versus predicted plots for VCC using (A) linear correlation of single‐frequency capacitance data and (B) O‐PLS model based on frequency scanning dielectric spectroscopy data. (C) Plot showing off‐line VCC data (black squares), single‐frequency (gray), and multifrequency online model data (blue). O‐PLS, Orthogonal‐Partial Least Squares; VCC, viable cell concentration.

These calibration data sets already suggest that dielectric spectroscopy data are superior for VCC monitoring compared to capacitance data that is generated at just a single frequency. This is supported in the literature [2, 10]. While lower cell concentrations occur during the initial growth phase of a perfusion process, higher VCCs are maintained by cell bleeding throughout the remaining culture duration. Decreased single‐frequency model performance at higher cell concentrations is related to variations in viable cell diameter that often increases over process time, likely attributed to changes in the metabolite concentrations and culture osmolality [11, 12, 13]. Due to the measurement principle of the dielectric spectroscopy probe, the single‐frequency signal increases not only with an increasing number of cells but also larger viable cell volume [14, 15]. In contrast, capturing dielectric properties at multiple frequencies enables much more detailed understanding of cell culture parameters [16, 17].

Both VCC models were run in parallel using data from a fresh perfusion cultivation. The data were not included in the initial calibration sets. The new data set was used to compare the two models’ prediction capabilities. In addition, both models were employed on‐line and the predicted VCCs were transferred to the process SCADA system in real‐time, therefore, fulfilling requirements for on‐line process control. Here, the nascent software that incorporates the O‐PLS model and acts as a middleware between the probe software and the SCADA system (see Figure 1), performed robustly and reliably throughout the processing time.

The evaluation of each model's prediction performance in comparison to the off‐line cell counter results is shown in Figure 2C. The predicted VCCs from both the single‐frequency and the multifrequency capacitance signals are very similar for the first 8 days of the process with RMSEP values of 5.04 and 5.13E6 cells/mL, respectively. Thus, the prediction accuracy noted during the exponential growth phase seen for the calibration data sets (see Figures 2A and B) was also noted with this new data set not used for O‐PLS model generation. As noted in the initial calibration data sets, the viable cell diameter increased over time in the new cultivation, here by more than 16% from day 7 to 10 [data not shown]. This resulted in an increased capacitance signal and therefore also predicted VCC for the single‐frequency model, even though the actual VCC was decreasing due to the start of the cell bleed as shown by the off‐line reference measurements. The cell diameter remained nearly constant from day 10 to the end of the process; whereby the single‐frequency prediction was always higher than the off‐line reference. This increased the RMSEP from 5.04E6 cells/mL in the initial growth phase to an overall prediction error of 7.75E6 cells/mL. In contrast, the multivariate model was able to maintain a high prediction accuracy throughout this transition with an overall RMSEP of 4.05E6 cells/mL for the 26‐day perfusion process. By utilizing dielectric spectroscopy data in combination with multivariate data analysis (MVDA), the VCC prediction error for this perfusion process was almost halved compared to traditional capacitance measurement at just one frequency. If only data from day 8 onwards are considered, the varying accuracies of both models become even more apparent, with average relative errors of 21% for the linear and only 7% for the multivariate model. For other processes where even stronger cell swelling may occur this difference in prediction accuracy would be even larger. In traditional fed‐batch processes, much higher differences in cell diameter can occur; which, in combination with other effects, makes dielectric spectroscopy the superior measurement principle [2]. By enabling robust on‐line VCC measurements and real‐time data transmission to control systems, the necessary requirements for complex process controls are provided.

Monitoring VCC in a mammalian cultivation process is critical for process statements and to take action for optimal process control. With the emergence of in‐line capacitance probes, an invaluable instrument for online determination of VCCs has found its way into the PAT toolbox. Although previous research articles highlighted the application of frequency scanning data and corresponding multivariate models for robust VCC determination in batch and fed‐batch processes, to our knowledge none of these models were deployed online for online determination of VCC. In this study, we were able to demonstrate the online setup of a self‐developed O‐PLS model and further integration into a SCADA system in an exemplary CHO perfusion cultivation. This approach allows online processing of multivariate raw data and the online viewing and integration of multivariate model results. It extends the application of multivariate models from merely post‐processing of data to online processing of data and direct integration into cultivation control software, which is essential for direct process control. We will continue our efforts to enable the online quantification of further parameters based on dielectric spectroscopy, such as cell diameter. Furthermore, we are aiming to streamline the presented approach into an easy‐to‐use software component capable of not only executing models for online processing of dielectric spectroscopy data, but also data from other multivariate data sources such as Raman spectroscopy systems.

## CONFLICT OF INTEREST STATEMENT

The authors declare no conflicts of interest with respect to the research, authorship, and/or publication of this article. All experiments have been done under Research and Development privilege and do not, to the best knowledge of the authors, stand in conflict to any third‐party rights.
